# Wharton’s jelly mesenchymal stem cells: a concise review of their secretome and prospective clinical applications

**DOI:** 10.3389/fcell.2023.1211217

**Published:** 2023-06-27

**Authors:** Hana Drobiova, Sardar Sindhu, Rasheed Ahmad, Dania Haddad, Fahd Al-Mulla, Ashraf Al Madhoun

**Affiliations:** ^1^ Human Genetics Unit, Department of Pathology, College of Medicine, Kuwait University, Jabriya, Kuwait; ^2^ Animal and Imaging Core Facilities, Dasman Diabetes Institute, Dasman, Kuwait; ^3^ Department of Immunology and Microbiology, Dasman Diabetes Institute, Dasman, Kuwait; ^4^ Department of Genetics and Bioinformatics, Dasman Diabetes Institute, Dasman, Kuwait

**Keywords:** WJ-MSCs, secretome, exosome, Wharton’s jelly mesenchymal stem cells, extracellular vesicles, EVs

## Abstract

Accumulating evidence indicates that most primary Wharton’s jelly mesenchymal stem cells (WJ-MSCs) therapeutic potential is due to their paracrine activity, i.e., their ability to modulate their microenvironment by releasing bioactive molecules and factors collectively known as secretome. These bioactive molecules and factors can either be released directly into the surrounding microenvironment or can be embedded within the membrane-bound extracellular bioactive nano-sized (usually 30–150 nm) messenger particles or vesicles of endosomal origin with specific route of biogenesis, known as exosomes or carried by relatively larger particles (100 nm–1 μm) formed by outward blebbing of plasma membrane called microvesicles (MVs); exosomes and MVs are collectively known as extracellular vesicles (EVs). The bioactive molecules and factors found in secretome are of various types, including cytokines, chemokines, cytoskeletal proteins, integrins, growth factors, angiogenic mediators, hormones, metabolites, and regulatory nucleic acid molecules. As expected, the secretome performs different biological functions, such as immunomodulation, tissue replenishment, cellular homeostasis, besides possessing anti-inflammatory and anti-fibrotic effects. This review highlights the current advances in research on the WJ-MSCs’ secretome and its prospective clinical applications.

## 1 Introduction

In recent years, the biological and clinical interest in mesenchymal stem cells (MSCs) has grown remarkably due to their distinctive stemness characteristics. MSCs are multipotent non-hematopoietic cells that exhibit high degree of self-renewal, multi-lineage differentiation potential and immunomodulatory activity (Pittenger et al., 1999; Ali et al., 2015).

MSCs reside primarily in the bone marrow, where they were first characterized; nevertheless, they have a broad post-natal organ distribution (Friedenstein et al., 1970). MSCs have been isolated from different adult and fetal tissues (Uder et al., 2018). The adult tissues include adipose tissue, skeletal muscle, bone marrow, molar teeth/dental pulp, synovium/synovial fluid, skin, hematopoietic supportive stroma, and others (da Silva Meirelles et al., 2006). The fetal tissues include peripheral and umbilical cord blood, umbilical cord stroma or tissue, placenta, amniotic fluid, endometrium (da Silva Meirelles et al., 2006; Jiang et al., 2011). Although, MSCs share common characteristics including the expression of common cell surface markers (CD105, CD73 and CD90) and multipotency capacity to differentiate into osteoblasts, chondrocytes, or adipocytes (Carvalho et al., 2011; Ghaneialvar et al., 2018), they have different expression profiles and properties.

The unique properties of Wharton’s Jelly (WJ)-MSCs attracted the attention of scientific community as an alternative source of stem cells for regenerative medicine. Unlike embryonic stem cells, no ethical concerns are associated with WJ-MSCs clinical application. Remarkably, both cell types have comparable molecular signatures as depicted from genetic profiling studies (Hsieh et al., 2010). Worth mentioning, umbilical cord blood MSCs share similar characteristics to that of WJ-MSCs, however, they are less attractive for clinical application due to their low frequency, poor proliferation rate and culture limitations (Zeddou et al., 2010).

WJ-MSCs characteristics qualify them as a better alternative for clinical use since WJ-MSCs are isolated from the gelatinous layer of the umbilical cord tissue using a non-invasive and painless procedure. Moreover, the umbilical cord is deemed a medical waste eliminating ethical concerns for their use (Kim et al., 2013). Thus, the use of WJ-MSCs overcomes the clinical limitations associated with adult MSCs such as the invasive collection procedures and the availability of suitable cell donors (Ali et al., 2015). Because of the embryonic nature of WJ-MSCs, the expression of the pluripotency markers, NANOG, Oct 3/4 and Sox2, is higher than that of the adult MSCs (Nekanti et al., 2010; Higuchi et al., 2012), and also implies less exposure to environmental toxins and associated genetic modulation which, may in part, explain their superiority over the adult MSCs (Fong et al., 2011). In comparison to adult MSCs, WJ-MSCs have a higher proliferation rate, longevity, differentiation potential, immune-privilege, and lower immunogenicity properties (Kim et al., 2013). Together, these advantages enable the use of WJ-MSCs as therapeutic agents in regenerative medicine. Notably, several clinical trials have been established to investigate the safety and efficacy of treatment with allogeneic WJ-MSCs (Uder et al., 2018; Carlsson et al., 2023). Yet, there are critical issues including heterogenicity as depicted from single cell transcriptomic studies (Chen et al., 2023), lack of clinical longitudinal studies addressing the long-term safety and prospective adverse conditions such as potential tumorigenicity, profibrogenicity, which were reported using adult MSCs (Russo et al., 2006; Barkholt et al., 2013). Together, these complications may add some complexity to their clinical applications.

In general, it was initially believed that the therapeutic effects of transplanted MSCs were facilitated by the migration of the cells to sites of injury, where they integrated into the damaged tissue and differentiated into specialized cells. But only a small number of cells were detected to engraft and survive in the damaged host tissue. Therefore, it became evident that the transplanted MSCs do not necessarily need to come in proximity with the damaged tissue. A growing body of evidence supports that the therapeutic effects of MSCs occur largely through paracrine signaling of secretome (Fong et al., 2014), which is classified into soluble factors (growth factors, cytokines, chemokines, and enzymes) and extracellular vesicles (EVs) such as exosomes and microvesicles (MVs) that additionally contain lipids, proteins, RNA and DNA subtypes (Daneshmandi et al., 2020). Therefore, delineating the secretome components and properties may assist with improving the therapeutic potential of MSCs (Nooshabadi et al., 2018). In this review, we discuss the WJ-MSCs’ secretome components compared to the secretomes of other MSCs as well as the therapeutic applications of these cells and their secretome in different disease conditions.

## 2 WJ mesenchymal stem cell’s origin and isolation

**FIGURE 1:**
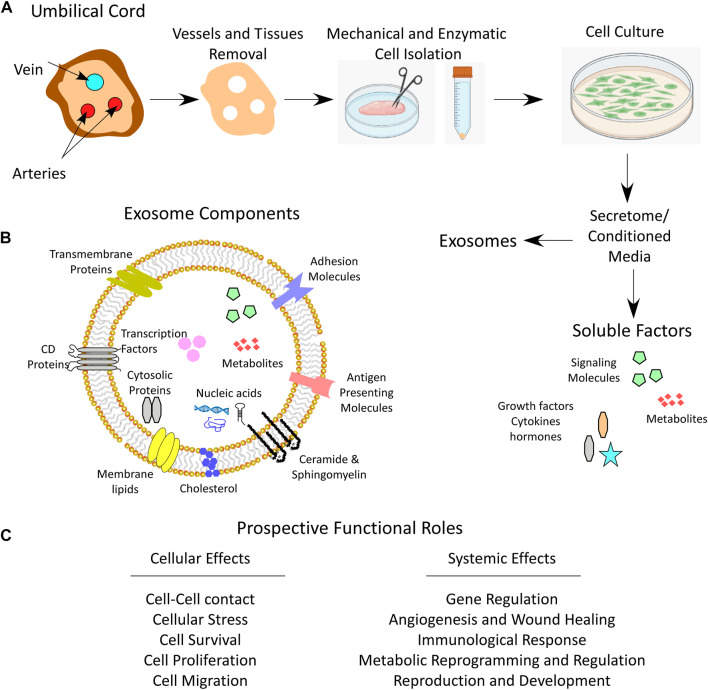
Umbilical cord WJ-MSCs and secretome. **(A)** Anatomical illustration of a cross section of umbilical cord depicting Wharton’s jelly, the process of WJ-MSCs mechanical and enzymatic isolation, culturing and secretome collection which contains both soluble and exosome fractions. **(B)** Schematic image for the exosome components. **(C)** Prospective functional roles of the secretome that influence cell function and system homeostasis.

During pregnancy, the umbilical cord forms a link between the mother and the fetus. From the outside, the umbilical cord is covered by a layer(s) of squamous-cubic epithelial cells, called umbilical epithelium (Can and Karahuseyinoglu, 2007; Wang et al., 2008). From the inside, the umbilical cord is composed of two arteries and one vein that are surrounded by a matrix of embryonic mucous connective tissue called WJ, which lies between the covering amniotic epithelium and the umbilical vessels (Figure 1) (Can and Karahuseyinoglu, 2007).

WJ’s function is to protect the enclosed vessels from compression, torsion and bending to maintain the blood flow between the fetal and maternal circulations. The mucous connective tissue contains specialized fibroblast-like cells and some mast cells. These stromal cells are called myofibroblasts because they exhibit some ultrastructural features of both smooth muscle cells and fibroblasts (Karahuseyinoglu et al., 2007). WJ is the major source of MSCs from the umbilical cord due to the large number of MSCs that may reach up to 4,700,000 MSCs/cm of the umbilical cord (Subramanian et al., 2015). In addition, the cells isolated from WJ show specific characteristics of MSCs, such as pluripotency and self-renewal as well as the ability to adhere to plastic in culture, the expression of specific surface antigens, namely CD105, CD73 and CD90, as well as their ability to differentiate into osteoblasts, adipocytes and chondroblasts (Ali et al., 2015).

### 2.1 Origin of WJ-MSCs

Although the ontogeny of MSCs is well-documented in both human and rodent fetal and adult tissues, little is known about the origin of WJ-MSCs. However, it is widely accepted that WJ-MSCs and adult MSCs have common parental cells, since both have similar structure and shape, possess the same surface markers, and have similar plasticity and multipotency (Can and Karahuseyinoglu, 2007). At the human embryonic stage E26-E27 [E11-E12 in mice (Mendes et al., 2005)], mesenchymal progenitor/stem cells initially arise in unique structures within the intra-embryonic aorta-gonad-mesonephros (AGM) region, i.e. in the earliest hematopoietic-forming sites (Wang et al., 2008). Although the hemangioblast compartments provide a good niche for the maintenance and proliferation of the mesenchymal progenitor/stem cells, these cells are different from their neighboring hematopoietic or endothelial progenitor cells (Durand et al., 2006; Guillot et al., 2007). In addition, WJ-MSCs are capable of proliferation and differentiation independently from any support by neighboring cells, whereas the hematopoietic stem cells (HSCs) are dependent on stromal cells as feeder cells. (Oostendorp et al., 2002; Mendes et al., 2005). During embryogenesis, MSCs co-localize with hematopoietic stem and progenitor cells, and circulate from the AGM region to various tissues (Migliaccio et al., 1986; Takashina, 1987; Zanjani et al., 1993; Tavian et al., 1999). Campagnoli et al., (2001) and Guillot et al., (2007) recovered a large number of MSCs from human fetal blood, liver, and bone marrow in the first-trimester of pregnancy, which showed the expression of pluripotency markers, demonstrated rapid growth and increased telomere length. However, in the second- and third-trimesters, the detected frequency of MSCs was low in the circulation and hematopoietic tissues, but high in the bone marrow, suggesting that MSCs undergo a migration process and are eventually stored in the bone marrow (Campagnoli et al., 2001). During the migration of MSCs from the AGM region to the fetal liver and bone marrow, some cells get trapped, and thus colonize the gelatinous material of the WJ, forming WJ-MSCs (Mendes et al., 2005; Batsali et al., 2013).

### 2.2 Isolation and culture of stromal cells

Cells from the umbilical cord can be isolated using two different methods, the explant method, or the enzymatic digestion method (Figure 1). The explant method requires mechanical tissue mincing that is followed by placing the tissue at substrate/tissue interface, which results in cell outgrowth on a plastic surface. The enzymatic digestion method, on the other hand, involves an additional step of tissue enzymatic digestion before plating on tissue culture plates (Mushahary et al., 2018). To isolate WJ-MSCs by enzymatic method, a freshly removed 5–10 cm long umbilical cord needs to be immediately transported to the laboratory in a sterile and cooled transfer medium (e.g. Hanks’ balanced salt solution). Then, before further processing of the tissue, arteries and veins are aseptically removed. After that, the cord is mechanically chopped and can be digested using enzymes such as collagenase, hyaluronidase, caseinase, clostripain and tryptic activity (Can and Karahuseyinoglu, 2007). The tissue homogenate is then filtered through 70–100 µm pore sized sieves to remove unnecessary tissue debris and the cells are plated, displaying a fibroblast-like appearance over the first culture period until the first passage (Figure 1) (Can and Karahuseyinoglu, 2007; Karahuseyinoglu et al., 2007).

### 2.3 Proliferation and senescence

For cell-based therapy, using adult MSCs involves some challenges, including also their failure to proliferate infinitely. They have a limited number of population doublings before they become senescent, that is a state of cell division arrest which eventually limits their immunomodulatory and differentiation capacities and thus, their clinical application is impeded (Fan et al., 2011; Turinetto et al., 2016). Due to their embryonic origin, WJ-MSCs show a delayed progression to senescence, compared to other MSCs (Batsali et al., 2017; Liau et al., 2020). Liau et al., (2020) observed no significant differences in WJ-MSCs’ proliferation, cell cycle, phenotype, and stemness marker expression after serial cell passaging. However, the expression of senescence-related gene, p21, and oncogene, c-Myc, was significantly upregulated at late passages (>20 cell passages). Furthermore, at low (<10) cell passages, WJ-MSCs adopt small fine-spindle shape which then transforms into flat, long, and broader cell morphology at later passages associated with low proliferation rate (Panwar et al., 2021). The late passage cells are non-tumorigenic, show slow cellular aging and do not exhibit chromosomal abnormalities. However, further passages demonstrate shorter telomere length (Panwar et al., 2021). Due to their embryonic nature, WJ-MSCs have low senescence rate relative to adult MSCs. Therefore, earlier passages of WJ-MSCs are good candidates for therapeutic use.

## 3 Principle of cell-fee based therapy

Several studies have demonstrated promising results for the treatment of different diseases using MSC-based therapy (Connick et al., 2012; Karantalis et al., 2014; Rushkevich et al., 2015; Vega et al., 2015; Fernández et al., 2018). Although the exact mechanism of action of MSCs remains unclear, various studies show that it is the secreted factors and EVs, collectively called the secretome, that cause the improvement rather than cellular differentiation at the site of injury or tumor *per se* (Gomes et al., 2018). The term secretome was originally defined by Tjalsma et al., (2000) as “both the components of machineries for protein secretion and the native secreted proteins.” However, currently the secretome is defined as “the factors that are secreted by a cell, tissue, or organ to the extracellular space at a specific time and under defined conditions” (Hathout, 2007; Agrawal et al., 2010). As mentioned above, the secretome is composed of soluble factors (growth factors, cytokines, chemokines, interleukins, prostaglandins, angiogenic mediators, hormones) and EVs including exosomes and MVs that harbor the vital molecules including lipids, proteins (cell adhesion molecules, extracellular matrix proteins, receptors, enzymes, metabolites, transcription factors), RNA and DNA subtypes inside or on their surfaces (Baraniak and McDevitt, 2010; Vizoso et al., 2017; Witwer and Théry, 2019; Daneshmandi et al., 2020; Xunian and Kalluri, 2020; Al Madhoun et al., 2021).

The use of cells’ secretome as a whole or only the EVs for treatment of diseases is termed as cell-free based therapy. Its benefits include the overcoming of ethical issues associated with cellular transplantation and preventing survival or complications resulting from incorrect differentiation of the cells in the host tissue, while maintaining the therapeutic potential (Chronopoulos and Kalluri, 2020; Kalluri and LeBleu, 2020).

## 4 Extracellular vesicles (EVs), their origin, subtypes, and composition

EVs are lipid bound vesicles harboring proteins, lipids and nucleic acids (Zaborowski et al., 2015; Bebelman et al., 2018) that are secreted into the extracellular space (Yáñez-Mó et al., 2015; Zaborowski et al., 2015; Théry et al., 2018). They play a role in intercellular communication and have the potential to alter the function of the recipient cell (White et al., 2006; Harding et al., 2013; Zaborowski et al., 2015). There are three principal subtypes of EVs including microvesicles (MVs), exosomes, and apoptotic bodies, which are distinguished based on their biogenesis and release pathways, their size, content, and function (Borges et al., 2013; Yáñez-Mó et al., 2015; Zaborowski et al., 2015). Despite the fact that their protein profiles vary based on their formation pathways, there are no specific distinguishing protein markers identified as yet. Exosomes are vesicles (30–150 nm in diameter) that are enclosed within a single outer membrane, originate from the endosome, and are secreted by all types of cells (Yáñez-Mó et al., 2015; Bebelman et al., 2018). Exosomes play a role in intercellular communication, cell maintenance, and tumor progression. They may also induce immune responses by acting as antigen-presenting vesicles (Bobrie et al., 2011; Chaput and Théry, 2011; Doyle and Wang, 2019). Microvesicles (MVs), also known as ectosomes, microparticles or shedding MVs, on the other hand, are vesicles (100 nm to 1 µm in diameter) (Borges et al., 2013; Yáñez-Mó et al., 2015; Zaborowski et al., 2015) that form by direct outward budding or pinching of the cell’s plasma membrane. It is believed that their formation requires cytoskeleton components (actin and microtubules), molecular motors (kinesins and myosins), and fusion machinery (SNAREs and tethering factors) (Cai et al., 2007). Due to their outward blebbing from the plasma membrane, in contrast to exosomes, MVs are abundant in cytosolic and plasma membrane associated proteins (Doyle and Wang, 2019), such as cytoskeletal proteins, integrins, heat shock proteins (HSPs), and tetraspanins (Willms et al., 2018). Annexin A1 which belongs to the family of Ca^2+^-dependent phospholipid-binding membrane proteins has been identified as a specific marker of MVs (Jeppesen et al., 2019). In mammals, MVs can be released by almost all cell types such as blood cells (platelets, leukocytes, and erythrocytes) (Wolf, 1967), endothelial cells (Elsner et al., 2023), and vascular smooth muscle cells (Boulanger et al., 2006). Apart from differences in their size and biogenesis, MVs and exosomes express different surface molecules used as biomarkers for their identification (Wu et al., 2013). Prototypic exosome markers include tetraspannins (CD9, CD63 and CD81) and ESCRT proteins (Alix and TSG101) (Hessvik and Llorente, 2018; Mathieu et al., 2021) while MVs are well studied in tumor cells and their markers frequently include CD40, ARF6, selectins, and flotillin-2 (Sedgwick and D'Souza-Schorey, 2018). MVs, like exosomes, are involved in intercellular communication. Apoptotic bodies (50 nm up to 5,000 nm in diameter) are released by dying cells due to separation of the plasma membrane from the cytoskeleton (Wickman et al., 2012). Unlike both exosomes and MVs, apoptotic bodies may contain intact organelles, chromatin, and small amounts of glycosylated proteins (Thery et al., 2001).

## 5 Wharton’s jelly MSCs secretome

### 5.1 Comparison of WJ-MSC’s secretome to that derived from other MSCs

The ability of the secretome to mediate various biological functions prompted exploratory studies on its use in cell-free therapies. The secretome of MSCs displays heterogeneous profiles depending on factors such as host age, source of MSCs, and the cell culture/differentiation media used (Paliwal et al., 2018). Investigating the differences in MSCs’ secretome and elucidating the mechanisms of action of their components may potentially facilitate effective and cell-free use of the secretome for treating different diseases (Kupcova Skalnikova, 2013; Driscoll and Patel, 2019; Kandoi et al., 2019; Wang L-T. et al., 2021; Munoz-Perez et al., 2021; Sandonà et al., 2021; Muzes and Sipos, 2022; Ghasemi et al., 2023).

Moreover, recent advances in analytical techniques have allowed the mapping of MSCs’ secretome and identifying the therapeutic factors applicable in regenerative medicine. The proteomic methods used for characterizing the secretome of MSCs are based on approaches involving immunological, shotgun and proteomic assays (Lavoie and Rosu-Myles, 2013). Immunological assays, including enzyme-linked immunosorbent assay (ELISA), Luminex antibody bead-based array, microarray, Western blotting, and cytokine antibody array, are highly specific, sensitive, and reproducible. While, the shotgun-based proteomics, two-dimensional gel electrophoresis, liquid chromatography with tandem mass spectrometry, stable isotope labeling by amino acids in cell culture (SILAC), matrix-assisted laser desorption/ionization time of flight (MALDI-TOF), MS/MS and quadrupole time-of-flight mass spectrometry (QTOF-MS), enable the identification of unknown and uniquely secreted proteins (Kandoi et al., 2019).

Significant differences in the secretomes’ profiles of MSCs from different sources have been documented (Shin et al., 2021). Moreover, Kim *et al.* observed a donor-to-donor variation in the secretome profiles of WJ-MSCs, even under identical culture conditions and passage number (Kim et al., 2019). Therefore, it is important to analyze the composition and functions of the secretome of different MSCs as it may affect their therapeutic potential. Hitherto, the best characterized secretome are those of bone marrow derived MSCs and adipose stem/stromal cells (Kupcova Skalnikova, 2013). Only recently, a comparative analysis of human WJ-MSC secretome has revealed the presence of a large number of proteins (Shin et al., 2021). For example, a study showed that alpha-2-macroglobulin (α2M) was the most highly expressed protein, after serum albumin (Bakhtyar et al., 2018). The secretome of these cells was also found to be enriched with cytokines/chemokines and growth factors, including interleukin (IL) 1-alpha (IL-1α), IL-1β, IL-6, IL-8, and granulocyte-macrophage colony-stimulating factor (GM-CSF), which was shown to have both pro- and anti-tumorigenic effects (Mirabdollahi et al., 2019). Other secreted factors include IL-2, IL-7, IL-12, IL-15, monocyte chemoattractant protein-1 (MCP-1), macrophage inflammatory protein-1beta (MIP-1β), regulated upon activation, normal T cell expressed and presumably secreted (RANTES), and platelet-derived growth factor (PDGF)-AA (Mirabdollahi et al., 2019). These factors are involved in cellular proliferation and differentiation, tissue remodeling, and regulating inductive events in patterning and morphogenesis; while chemoattractants such as MCP1, MIP-1β, RANTES, hepatocyte growth factor (HGF), fibroblast growth factor-2 (FGF-2), and PDGF-AA, facilitate mobilizing of immune cells in the process (Yoo et al., 2009; Prasanna et al., 2010; Konala et al., 2020). A recent study that compared expression profiles of WJ-MSCs and bone marrow derived MSCs reported significant differences between both (Barrett et al., 2019). They found that 436 genes were significantly and differentially expressed in WJ-MSCs (Barrett et al., 2019). These genes play a role in different processes, such as immunomodulation, angiogenesis, wound healing, apoptosis, antitumor activity, and chemotaxis (Barrett et al., 2019). The authors are suggesting that these differences may explain the advantages of using WJ-MSCs over BM-MSCs in clinical applications (Barrett et al., 2019). A myriad of biomolecules and factors detected in the secretome of different MSCs is summarized in Table 1.

**TABLE 1:** Comparison of the components of different MSCs’ secretome.

Function	Marker	WJ-MSCs^a^	AD-MSCs^b^	BM-MSCs^c^	DP-MSCs^d^	Peripheral MSCs^e^
Angiogenesis	ANG		✓	✓		
	ANGPT1	✓	✓	✓	✓	
Chemokine	CCT8	✓				
	CCL5	✓		✓		
	MCP1	✓	✓			
	MIP-1B	✓				
	SDF-1	✓		✓	✓	
Cytokine	IFN-g	✓		✓	✓	
	IL-10	✓		✓	✓	
	IL-12	✓				
	IL-15	✓				
	IL-1a	✓	✓	✓		
	Il-1b	✓	✓			
	IL-2	✓				
	IL-4		✓	✓		
	IL-6		✓	✓	✓	✓
	IL-7	✓				
	IL8	✓	✓			
Cytoskeleton	ACTA2	✓	✓	✓	✓	✓
	ACTB	✓	✓	✓	✓	✓
	ACTC1	✓	✓	✓	✓	✓
	ACTG2	✓				
	ACTN4	✓	✓			
	DES	✓				
	FLNA	✓				
	SPTA1	✓				
	SPTB	✓				
	TAGLN	✓				
	TPM2	✓				
	TUBB	✓				
	VIM	✓	✓			
ECM protein	FBN1	✓	✓			
	FN1	✓	✓			
Enzyme	GAPDH	✓				
	IDO					✓
Functional protein	MYH11	✓				
	MYH14	✓				
	MYH9	✓				
	MYL6	✓				
	TLN1	✓				
Growth factor	FGF-2	✓	✓	✓	✓	
	GDF6			✓		
	GM-CSF	✓				
	HGF	✓	✓		✓	
	NGF				✓	
	PDGF-1		✓	✓	✓	
	TGF-B	✓	✓	✓	✓	
	VEGF	✓	✓	✓	✓	✓
Hemoglobin	HBA1	✓				
	HBB	✓				
	HBG2	✓				
Hormone	IGF-1	✓	✓	✓		✓
Immune system	IGHG2	✓				
	IGHG3	✓				
	IGHM	✓				
	IGKC	✓				
	IGLC2	✓				
Inflammation	ANXA1	✓				
Inhibitor	TIMP2		✓	✓		✓
lipid metabolism	APOA1	✓				
Membrane skeletal protein	ANK1	✓				
Nucleoprotein	AHNAK	✓				
plasma membrane protein	SLC4A1	✓				
Plasma protein	FGB	✓				
	A2M	✓				
	ALB	✓	✓	✓	✓	✓
	C3	✓				
	TF	✓				
Pleiotropic protein	ANXA2	✓				
Prostaglandin	PGE2	✓				
Ribosomal protein	RPLP2	✓				
Serine protease inhibitor	SERPINA1	✓	✓	✓		
References		Bakhtyar et al., (2018), Mirabdollahi et al., (2019), Konala et al., (2020), Yoo et al., (2009); Prasanna et al., (2010)	Chang et al., (2017), Mussano et al., (2017), An et al., (2021), Chen et al., (2022)	Oskowitz et al., (2011), Katagiri et al., (2017), Baberg et al., (2019), Konala et al., (2020)	Rajan et al., (2017), Konala et al., (2020)	van Buul et al., (2012), Katagiri et al., (2016)

A2M, Alpha-2-Macroglobulin; ACTA2, Actin; aortic smooth muscle; ACTB, Actin; cytoplasmic 1; ACTC1, Actin; alpha cardiac muscle; ACTG2, Actin; gamma-enteric smooth muscle; “ACTN4, Actinin Alpha 4”; AHNAK, Desmoyokin, ALB, Albumin; ANG, Angiogenin; ANGPT1, Angiopoietin-1; ANK1, Ankyrin 1; ANXA1, Annexin A1; ANXA2, Annexin A2; APOA1, Apolipoprotein A1; C3, Complement C3; CCL5, RANTES; CCT8, Chaperonin Containing T-Complex Polypeptide 1 Subunit 8; DES, Desmin; FBN1, Fibrillin 1; FGB, Fibrinogen Beta Chain; FGF-2, Fibroblast growth factor 2; FLNA, isoform 2 of filamin-A; FN1, Fibronectin 1; GAPDH, Glyceraldehyde-3-Phosphate Dehydrogenase; GDF6, Growth Differentiation factor 6; GM-CSF, Granulocyte-Macrophage Colony-Stimulating Factor; HBA1, Hemoglobin Subunit Alpha 1; HBB, Hemoglobin Subunit Beta; HBG2, Hemoglobin Subunit Gamma 2; HGF, Hepatocyte Growth factor; IDO, Indoleamine 2; 3-Dioxygenase 1; IFN-g, Interferon g; IGF-1, Insulin-like growth factor-1; IGHG2, Immunoglobulin Heavy Constant Gamma 2; IGHG3, Immunoglobulin Heavy Constant Gamma 3; IGHM, Immunoglobulin Heavy Constant Mu; IGKC, Immunoglobulin Kappa Constant; IGLC2, Immunoglobulin Lambda Constant 2; IL-10, Interleukin-10; IL-12, Interleukin-12; IL-15, Interleukin-15; IL-1a, Interleukin-1a; Il-1b, Interleukin-1b; IL-2, Interleukin-2; IL-6,; IL-7, Interleukin-7; IL8, Interleukin-8; MCP1, Monocyte Chemoattractant Protein-1; MIP-1B, Macrophage Inflammatory Protein 1-Beta; MYH11, Isoform 2 of Myosin 11; MYH14, Myosin Heavy Chain 14; MYH9, Myosin Heavy Chain 9; MYL6, Myosin Light Chain 6; NGF, Nerve Growth Factor; PDGF-1, Platelet Derived Growth Factor Subunit A; PGE2, Prostaglandin E2; RPLP2: Ribosomal Protein Lateral Stalk Subunit P2, SDF-1: Stromal Cell-Derived Factor 1; SERPINA1, Serpin Family A Member 1; SLC4A1, Solute Carrier Family 4 Member 1; SPTA1, Spectrin Alpha; Erythrocytic 1; SPTB, Spectrin Beta Chain; Erythrocytic; TAGLN, Transgelin; TF, Transferrin; TGF-B, Transforming growth factor b; TIMP2, Tissue Inhibitor Of Metalloproteinases 2; TLN1, Talin 1; TPM2, Tropomyosin 2; TUBB, Tubulin Beta Class I; VEGF, Vascular Endothelial Growth Factor; VIM, Vimentin.

^a^ Wharton’s jelly mesenchymal stem cells.

^b^ Adipose tissue mesenchymal stem cells.

^c^ Bone marrow mesenchymal stem cells.

^d^ Dental pulp mesenchymal stem cells.

^e^ Peripheral blood mesenchymal stem cells.

The ✓sign implies the existence of the marker.

### 5.2 Therapeutic potential and applications of WJ-MSCs and their secretome

As mentioned above, it is thought that MSCs facilitate the tissue and organ repair by their multipotent potential that enables them to replace the damaged cells (Mahmood et al., 2003; Murphy et al., 2003). However, it was later suggested that in response to tissue injury, MSCs home to the damaged site and stimulate repair by producing trophic factors such as growth factors, cytokines, and antioxidants (Chen et al., 2008; Karp and Leng Teo, 2009). Some of these factors impart MSCs their immunomodulatory potential (English et al., 2010). In general, the biological characteristics of MSCs that form the basis of their clinical applications include: (a) their ability to home to sites of inflammation following tissue injury when injected intravenously (Rustad and Gurtner, 2012); (b) the secretion of multiple bioactive molecules capable of stimulating recovery of injured cells and inhibiting inflammation (Ranganath et al., 2012), (c) modulating the immune functions (Lo Iacono et al., 2018), (d) differentiation into various cell types (Wang S. et al., 2012), and (e) as a tool for gene therapy (Kamal and Kassem, 2020).

Because the secretome of WJ-MSCs plays roles in cellular homeostasis, anti-inflammation, tissue replenishment, immunomodulation, and other functions (Tang et al., 2021), the therapeutic potentials of WJ-MSCs and their secretome have been explored for several disease conditions as briefly reviewed in the following sections.

### 5.3 Immunomodulatory properties

The clinical utility of WJ-MSCs is tantamount, due basically to their low immunogenicity (Liu et al., 2012; Kim et al., 2013; Varaa et al., 2019). WJ-MSCs were found to express low-to-moderate levels of MHC class I (HLA-ABC) molecules (Prasanna et al., 2010; Liu et al., 2012; de Girolamo et al., 2013) and lack the expression of MHC class II (HLA-DR) and co-stimulatory antigens such as CD40, CD80 and CD86 that lead to T- and B-cell mediated responses (Pappa and Anagnou, 2009; Prasanna et al., 2010). Their immunosuppressive potential also relates to their ability to produce large quantities of immunosuppressant cytokines, such as TGF-β, IL-10, and VEGF (Weiss et al., 2008).

Interestingly, MSCs, including WJ-MSCs, may interact with and modulate the activation and function of all key immune effector cells including T or B cells (Le Blanc et al., 2004; Aggarwal and Pittenger, 2005; Uccelli et al., 2006; Carreras-Planella et al., 2019), monocyte or macrophages (Cutler et al., 2010; Dymowska et al., 2021; Lu et al., 2021), dendritic cells (DCs) (Tipnis et al., 2010; Gao et al., 2017; Vieira Paladino et al., 2019), neutrophils (Khan et al., 2015; Ahn et al., 2020; Taghavi-Farahabadi et al., 2021), mast cells (Brown et al., 2011; Cho et al., 2022), and natural killer cells (Casado et al., 2013; Najar et al., 2018; Abbasi et al., 2022). Although the mechanism of immunomodulatory activity remains to be elucidated, it is thought that both cell-to-cell contact and soluble factors are the key players in WJ-MSCs mediated immunosuppression (Shi et al., 2010; Ma et al., 2014). Of note, WJ-MSCs and their secretome possess the immunomodulatory properties (Mrahleh et al., 2021; Muzes and Sipos, 2022), in addition to exerting anti-inflammatory effects (Munoz-Perez et al., 2021). In this regard, immune-modulatory effects of WJ-MSCs secretome were related to the presence of several secreted factors, including IL-2, IL-6, IL-12, IL-15, CXCL8 (IL-8), CCL2 (MCP-1), CCL3/4 (MIP-1), CCL5 (RANTES), and prostaglandin-E2 (PGE2) (Yoo et al., 2009). Taghavi-Farahabadi et al., (2021) demonstrated that WJ-MSCs’ secretome improved the function and expanded the lifespan of neutrophils, which might have therapeutic applications for treating neutropenia or chronic granulomatous disease. These positive effects of exosomes were ascribed to miRNAs and mRNAs, as well as several secreted factors present in exosomes, including tumor necrosis factor α (TNFα), G-CSF, interferon (IFN)-γ, IFN-α, IL-8, and IL-6 (Taghavi-Farahabadi et al., 2021). Moreover, WJ-MSCs can modify T cell receptor-mediated T cell activation via EVs enriched with programmed death-ligand 1 (PD-L1) which reduces T cell activation in acute graft *versus* host disease (Li et al., 2021). WJ-MSCs’ exosomes also proved to be beneficial for treating lymphedema by increasing the expression of lymphangiogenic factors including angiopoietin-2 (Ang2), prospero-homeobox protein 1 (Prox1), and phospho-Akt (Ting et al., 2021). Moreover, based on their immunomodulatory effects, whether through cell-to-cell contact or soluble factors, WJ-MSCs and their secretome have been used to successfully treat morbid conditions, such as graft *versus* host disease (Newell et al., 2014; Soder et al., 2020; Pochon et al., 2022), diabetes (Katuchova et al., 2015; Moreira et al., 2017; Gao et al., 2018; Qi et al., 2019), and cancer (Hendijani et al., 2015).

### 5.4 Tissue repair and injury prevention

#### 5.4.1 Tissue repair

Tissue repair is defined as a compensatory regeneration and restoration of tissue architecture and function following a surgical, mechanical, or chemical-induced injury (Krafts, 2010). Tissue repair is a dynamic complex process that involves the coordinated action of many different cells and molecules. The mechanism of tissue repair includes the activation of immune response, angiogenesis, innervation, epithelialization, and scar formation, best reviewed in (Deng et al., 2022) and (Eming et al., 2014). Notably, the administration of WJ and other sources MSCs’ secretome improves the tissue repair due to its ability to modulate the process of inflammation by inducing anti-inflammatory responses (Chen et al., 2015; Hervás-Salcedo et al., 2021; Miyano et al., 2022; Wang et al., 2022), including also the M2 macrophage polarization (Nakajima et al., 2012; Zhou et al., 2016; Luz-Crawford et al., 2017; Wang J. et al., 2021). Furthermore, WJ-MSCs’ secretome was found to mediate angiogenesis, neuroprotection and neurogenesis (Hsieh et al., 2013).

#### 5.4.2 Wound healing and repair

Despite the efforts focused on wound care and new therapeutic approaches for acute and chronic wound management, wound healing is still a challenging clinical problem. The process of wound healing involves an interplay between several cell populations, the extracellular matrix and the action of soluble mediators including growth factors and cytokines. The process may be studied by dividing it into four phases, (i) coagulation and hemostasis, (ii) inflammation, (iii) proliferation, and (iv) wound remodeling with scar tissue formation, best reviewed in (Velnar et al., 2009).

The pioneering work by Bakhtyar et al., (2018) identified that the exosomes isolated from WJ-MSCs promote skin wound healing by increasing fibroblasts viability, migration, and the expression of myofibroblast marker alpha smooth muscle actin (αSMA) and enhanced skin wound healing in the punch biopsy wound model in mice. Proteomic analysis of exosomes revealed that the alpha-2-macroglobulin (α2M) protein played a key role in promoting wound healing (Bakhtyar et al., 2018). Similarly, another group of researchers also found that the exosomes of WJ-MSCs were instrumental in enhancing skin wound healing and the underlying mechanism involved attenuation of cell death by suppressing nuclear translocation of apoptosis-inducing factor (AIF) which is a mitochondrial oxidoreductase that contributes to cell death and participates in the respiratory chain assembly (Zhao et al., 2020). Kim et al., (2019) reported that the pro-angiogenic activities of WJ-MSCs were related to their secretome containing angiogenin, MCP-1, IL-8, and VEGF.

#### 5.4.3 Neuroprotection

Perinatal brain injury (PBI) is one of the main causes of perinatal morbidity and mortality (Volpe, 1995). PBI is mainly caused by cerebral ischemia, cerebral hemorrhage, or ascending intrauterine infection because of accidental trauma or genetic disorders. PBI has an enormous impact on the effected family and society which requires co-operation between physicians, neurologists, physio-, speech-, and psychotherapists, as well as other specialists. (Jensen et al., 2003). More effective neuroprotective strategies are being developed. One of these strategies involves the use of WJ-MSCs’ exosomes, such as to alleviate the pathogenesis of PBI which is associated with the death of neurons and pre-oligodendrocytes and by reducing microglia-mediated neuroinflammation (Thomi et al., 2019a). Thomi et al., (2019a) demonstrated that exosomes of WJ-MSCs exhibited the anti-inflammatory potential, both *in vitro* and *in vivo*, by targeting microglia cells which reduced the expression of pro-inflammatory cytokines through interference with the toll-like receptor 4 (TLR4)/CD14 pathway. The same group of researchers also reported that intranasal administration of WJ-MSCs’ exosomes could protect white and gray matter in PBI by improving neuron cell viability, development, and the recovery of learning ability in animal models of PBI (Thomi et al., 2019b).

Neuroprotective potential of WJ-MSCs’ secretome was also demonstrated in Alzheimer’s disease. Alzheimer’s disease is a progressive brain disease that negatively affects the performance of daily activities in older individuals. This progressive cognitive decline is associated with the accumulation of amyloid-beta (Aβ) and tau proteins (Selkoe and Hardy, 2016). The accumulation of Aβ, produced by sequential cleavage of amyloid precursor protein (APP) by beta-secretase and gamma-secretase, results in the formation of Aβ oligomers that are toxic to neurons (Haass and Selkoe, 2007). In contrast, tau protein results from alternative splicing of the microtubule associated protein tau (MAPT) gene, forming soluble protein isoforms (Goedert et al., 1989). Several functional interactions between these two proteins result in neural circuit damage and cognitive decline in Alzheimer’s disease. Unfortunately, no treatment that cures this disease is available yet. However, one of the recent treatment approaches is to explore the neuroprotective potentials of MSCs’ exosomes (Zhang et al., 2021; Kandimalla et al., 2023). EVs from WJ-MSCs were shown to protect against Alzheimer’s disease by preventing the damage caused by amyloid beta oligomers in hippocampal neurons (Bodart-Santos et al., 2019). WJ-MSCs’ exosomes also improved spatial memory in Alzheimer’s disease models of olfactory bulbectomized mice (Zhdanova et al., 2021). WJ-MSCs-conditioned media was reported to improve Schwann cell viability and proliferation (Guo et al., 2015). Similarly, hepatocyte growth factor (HGF) and brain-derived neurotrophic factor (BDNF) secreted by WJ-MSCs were found to have neuroprotective effects on the damaged neurons (Mukai et al., 2018).

#### 5.4.4 Anti-fibrotic potential of WJ-MSCs and their secretome

Fibrosis is defined as an overgrowth, hardening, and/or scarring of different tissues due to the formation and deposition of excess extracellular matrix components including collagen and fibronectin, leading to formation of scar tissue (Schuppan et al., 2001; Wynn, 2008; Wynn and Ramalingam, 2012). The resulting replacement of the normal tissue by fibrous tissue disrupts the structure and function of the tissue (Tomasek et al., 2002; Friedman, 2004; Wynn, 2007), causing an impaired function of the organ affected which may lead to life-threatening complications. Fibrotic diseases can affect different organs and tissues including the lung (pulmonary fibrosis), liver (liver cirrhosis), heart (cardiac fibrosis), kidney (renal fibrosis) and skin (systemic sclerosis) (Wernig et al., 2017).

Although it is believed that fibrosis is the end result of chronic inflammation (Wynn, 2008), accumulating evidence suggests that the mechanisms inducing fibrogenesis are different from those regulating inflammation (Wynn, 2008). Fibrosis is a complex and multifactorial process that may be triggered by different factors (Wynn, 2008). However, in all fibrotic diseases, fibrotic tissue remodeling begins by the activation of ECM-producing myofibroblasts (Gabbiani, 2003; Wynn and Ramalingam, 2012) that leads to the production of surplus quantities of extracellular matrix proteins comprising of 43 types of collagen subunits, 36 proteoglycans and about 200 types of complicated glycoproteins (Hynes and Naba, 2012). These myofibroblasts can develop from different sources including the resident mesenchymal cells, epithelial/endothelial-mesenchymal (EMT/EndMT) transition (Kalluri and Neilson, 2003; Quan et al., 2006; Willis et al., 2006; Zeisberg et al., 2007) or from fibrocytes that are derived from bone-marrow stem cells (Bucala et al., 1994). Autocrine factors secreted by myofibroblasts as well as different paracrine signals from lymphocytes and macrophages can activate myofibroblasts (Wynn, 2008; Van Linthout et al., 2014). In addition, the pathogen-associated molecular patterns (PAMPs) may also play a role in myofibroblast activation (Otte et al., 2003; Coelho et al., 2005; Meneghin and Hogaboam, 2007).

Currently, the treatment options for fibrotic diseases are limited (Rosenbloom et al., 2017) and mainly focus on symptom management and target the inflammatory response (Zhao et al., 2022). The multifactorial etiology and redundancy of pathways involved make it hard to find a single drug that will be successful in stopping or modifying fibrotic disease progression. Therefore, anti-fibrotic therapy development requires targeting the molecular pathways that lead to fibrosis including inhibiting the activation or proliferation of fibroblasts, promotion of excessive ECM degradation, or modulating the immune response (McVicker and Bennett, 2017). In this regard, research studies concerned with the development of anti-fibrotic therapies are reporting encouraging results (Zhao et al., 2022), even in cell-based therapies (Cheng et al., 2022).

The fact that WJ-MSCs possess immunomodulatory and anti-fibrotic properties attracted attention to their therapeutic potential. The anti-fibrotic potential of WJ-MSCs is multifactorial and may involve a combination of direct and indirect effects on the cellular and molecular mechanisms involved in fibrosis (Lin et al., 2010). The direct effects may include inhibition of fibroblast activation and proliferation, and the reduction of collagen synthesis and deposition in the extracellular matrix (Lin et al., 2010). WJ-MSCs’ secretome contains factors that can directly modulate these processes, including transforming growth factor β (TGF-β) inhibitors, matrix metalloproteases (MMPs) that degrade excess extracellular matrix and anti-inflammatory cytokines such as IL-10 that can reduce inflammation and tissue damage (Muzes and Sipos, 2022). The indirect effects of WJ-MSCs that contribute to their anti-fibrotic potential may involve their ability to modulate the immune response, stimulate tissue regeneration and repair and enhance angiogenesis (Ahangar et al., 2020). Tissue repair and regeneration is mediated by the ability of WJ-MSCs to differentiate into various cell types, such as fibroblasts, epithelial cells, and endothelial cells (Ali et al., 2015). Moreover, WJ-MSCs ability to enhance angiogenesis, which in turn improves tissue perfusion and oxygenation, stimulates healing and may play a role in the anti-fibrotic characteristics of these cells (Hsieh et al., 2013).

In contrast, WJ-MSCs may exert indirect effects on fibrosis by their ability to modulate the immune response, promote tissue regeneration, and enhance angiogenesis (Planat-Benard et al., 2021). The immunomodulatory activity of WJ-MSCs is mediated by their secretome which contains factors, such as TGF-β and IL-10, that regulate the activity and proliferation of immune cells eventually reducing pro-inflammatory cytokines production and inhibiting the immune response that leads to fibrosis (Planat-Benard et al., 2021). It was demonstrated that WJ-MSCs exhibit increased expression of immunosuppressive proteins, such as leukocyte antigen G6 (HLA-G6) that plays a vital role in avoiding immune-based responses against the embryo, indoleamine-2,3-dioxygenase (IDO), and PGE2 (Weiss et al., 2008). Preliminary results of clinical studies using WJ-MSCs’ secretome, on the other hand, have demonstrated promising anti-fibrotic potential in patients with liver cirrhosis (Ding et al., 2022), pulmonary fibrosis (Liu et al., 2020), and renal fibrosis (Di Vizio et al., 2012).

##### 5.4.4.1 Liver fibrosis

Liver fibrosis is a wound healing response to chronic injuries which if not treated can progress to liver cirrhosis (Suk and Kim, 2015; Liedtke et al., 2022). Although, numerous drugs were proven to have anti-fibrotic activity both *in vitro* and in animal models, none of them was effective for clinical use. Therefore, until now, the only effective therapy for end-stage liver disease remains the liver transplantation. Recently, research of liver disease treatment using MSCs is gaining attention, especially that studies have demonstrated the ability of human WJ-MSCs to differentiate into hepatocyte-like cells *in vitro* (Campard et al., 2008; Zhang et al., 2009; An et al., 2014).

Recent studies provide promising evidence for the use of WJ-MSCs in the treatment of liver fibrosis (Kao et al., 2020; Afarin et al., 2022). The suggested mechanisms of the therapeutic potential of WJ-MSCs regarding liver fibrosis include the paracrine effects, trans-differentiation into hepatocyte-like cells, and immunomodulatory functions (Liu et al., 2015).

The effect of WJ-MSCs on liver fibrosis has been assessed by several investigators (Tsai et al., 2009; Lin et al., 2010; Kao et al., 2020; Afarin et al., 2022). In rats, Tsai et al., (2009) have shown that injection of WJ-MSCs significantly reduced the liver fibrosis by decreasing collagen deposition, serum levels of glutamic oxaloacetic transaminase, glutamic pyruvate transaminase, and TGF-β1 and increasing mesenchymal-epithelial transition factor-phosphorylated type and hepatocyte growth factor.

Lin et al., (2010), on the other hand, investigated the use of WJ-MSCs in treatment of liver fibrosis using chemically induced liver fibrosis model. In this model, liver fibrosis was induced in rats via intraperitoneal injection of thioacetamide. WJ-MSCs were transplanted into liver-damaged rats via the portal vein and the effects were monitored by serum biochemistry and histopathology assessment and the authors found that WJ-MSCs transplantation significantly recovered serum prothrombin time and serum albumin was also improved (Lin et al., 2010). Collagen accumulation decreased after 14 days of transplantation and immunohistochemical analysis revealed that the transplanted WJ-MSCs produced albumin, HGF, and metalloproteinase (MMP), suggesting that WJ-MSCs might alleviate liver collagen and could be used in liver fibrosis therapy (Lin et al., 2010). Another study by Hammam et al., (2016) investigated the antifibrotic potential of combining either early or late WJ-MSCs treatment combined with praziquantel on both acute and chronic stages of *Schistosoma mansoni*-induced liver fibrosis in mice. Following transplantation, WJ-MSCs exhibited differentiation into functioning liver-like cells, which was proven by their expression of human hepatocyte-specific markers (Hammam et al., 2016). Regression of liver fibrosis was also evidenced by histopathological, morphometric, and gelatin zymographic results, in addition to the reduction of three vital contributors to liver fibrosis in the model studied including alpha smooth muscle actin, collagen-I, and interleukin-13 (Hammam et al., 2016). Praziquantel enhanced the benefits observed in the WJ-MSCs treated groups (Hammam et al., 2016). However, Rengasamy et al., (2017) indicated that CCl4-induced liver fibrosis was alleviated more effectively using human BM-MSCs than by WJ-MSCs in rat models. This could be explained by differential expression patterns of matrix metalloproteases and angiogenic factors produced by bone marrow and Wharton’s jelly derived MSCs.

It was also shown that paracrine activity of MSCs plays a role in tissue damage repair through exosomes (Salehipour Bavarsad et al., 2022). However, the types and concentrations of inflammatory mediators, such as TGF-β1 in the MSCs’ microenvironment may affect their function and therapeutic potential. In this concern, Salehipour Bavarsad et al., (2022) investigated whether WJ-MSCs pretreated with different concentrations of TGF-β1 change the anti-fibrotic properties of their exosomes on activated hepatic stellate cells. Their results demonstrated that exosomes isolated from untreated WJ-MSCs reduced TGFβ-smad2/3 signaling and expression of fibrotic markers. These effects were even more intense upon using exosomes derived from 0.1 ng/ml TGFβ-pretreated WJ-MSCs, suggesting that these pre-treated WJ-MSCs might significantly benefit the liver fibrosis patients (Salehipour Bavarsad et al., 2022).

##### 5.4.4.2 Pulmonary fibrosis

Pulmonary fibrosis is a chronic, progressive lung disease that is characterized by progressive lung scarring, eventually leading to respiratory failure and death (Martinez et al., 2017). There are currently only two anti-fibrotic agents, namely nintedanib (ofev) and pirfenidone (Esbriet) (Marijic et al., 2021), that are FDA-approved for treating idiopathic pulmonary fibrosis which is the most common form of pulmonary fibrosis and slow down the disease progression and scarring in the lungs, but also have multiple side effects and do not cure the disease (Marijic et al., 2021). Therefore, research is attracted to investigating the utility of anti-fibrotic characteristics of MSCs for the treatment of pulmonary fibrosis. The applicability of WJ-MSCs as an anti-fibrotic agent in lungs has been demonstrated in the following studies. For example, Periera-Simon et al., (2021) compared the therapeutic potential of different sources of MSCs including WJ-MSCs in the aging mouse model of bleomycin (BLM)-induced lung fibrosis. Their results showed that all sources of MSCs, except chorionic membrane cells (CSC), decreased the Ashcroft score [a pulmonary fibrosis evaluation procedure based on histological sample analysis (Hübner et al., 2008)] and hydroxyproline levels [collagen metabolism evaluation test (Qiu et al., 2014)] on day 10 after infusion into the BLM-treated mice. The observed phenotype was mainly due to a reduction in the gene expression of αv-integrin- and TNF-α, protein markers for fibrosis and inflammation, respectively; thus,, suggesting that WJ-MSCs could promote the repair of fibrotic lung tissue (Periera-Simon et al., 2021). Another study reported that WJ-MSCs repress inflammation, reduce myofibroblast action, and enhance MMP-9 and TLR-4 receptor expression, leading to alleviation of fibrosis (Chu et al., 2019). Moreover, in a small pilot study of patients with pulmonary fibrosis, WJ-MSCs infusion led to improved lung function and reduced fibrosis as assessed by imaging studies (Yang et al., 2021; Saleh et al., 2022a).

The therapeutic potential of WJ-MSCs was also tested for the treatment of coronavirus pandemic 2019 (COVID-19) (Saleh et al., 2021; Saleh et al., 2022b), caused by SARS-CoV-2 which is known to induce a severe cytokine storm in the lungs that causes edema, defective respiration, acute respiratory distress syndrome, acute heart damage, and secondary infections (Huang C. et al., 2020), and eventually death (Huang P. et al., 2020). Owing to their’ immunomodulatory property, WJ-MSCs were suggested to attenuate COVID-19 cytokine storms by suppressing T-lymphocytes (Aggarwal and Pittenger, 2005). WJ-MSCs play an important role in modulating immune system by secreting large amounts of anti-inflammatory cytokines such as IL-10, TGF-β, IL-6, and VEGF (Zhang et al., 2020; Saleh et al., 2021). The immunomodulatory secretome of MSCs is stimulated by the pathogen-related molecules including LPS and/or dsRNA of viruses that activate the TLR receptors on MSCs (Waterman et al., 2010; Li et al., 2012). MSCs secrete paracrine factors such as keratinocyte growth factor (KGF), Ang-1, PGE2, IL-10, and other trophic cytokines which eventually enhance the alveolar fluid clearance, regulate epithelial and endothelial permeability of the lung, promote endothelial repair, and reduce inflammation (Maron-Gutierrez et al., 2014). In critically severe-type COVID-9 patients, Zhang et al., (2020) demonstrated that WJ-MSCs intravenous injections improves pulmonary function, lung inflammation, and patients’ recovery within 7 days with no obvious adverse conditions. Similarly, a phase I clinical trial demonstrated the therapeutic potential of WJ-MSCs in COVID-19 patients. In this trial, patients received WJ-MSCs intravenous injections three times three days apart, which was sufficient to improve the immune system function as demonstrated by an increase in lymphocytes percentage, absolute lymphocyte count, and CD4 and CD8 T cell ratios (Saleh et al., 2021). Moreover, a 1-year follow up of these patients demonstrated that WJ-MSCs treatment did not cause any serious complications or tumor development (Saleh et al., 2022b). Currently, there are several ongoing clinical studies that may improve the understanding about the therapeutic potential of WJ-MSCs’ and their secretome in the therapy of COVID-19 (Harrell et al., 2020). Together, these results indicate that WJ-MSCs inhibit overactivation of the immune system caused by COVID-19 and promote endogenous repair by improving the microenvironment.

##### 5.4.4.3 Renal fibrosis

Hu et al., (2020) have shown that seeding the human WJ-MSCs into the decellularized kidney scaffold ameliorates the renal fibrosis through decreasing EMT by the TGF-β/SMAD signaling pathway following subtotal nephrectomy in rats. WJ-MSCs exhibited anti-fibrotic effects in unilateral ischemia-reperfusion injury rat model of renal fibrosis through the mechanism involving delayed epithelial-to-mesenchymal transition and reduced renal fibrosis (Du et al., 2013). Thus, WJ-MSCs hold a promising potential for the treatment of fibrotic diseases. However, more research is required to better understand their mechanisms of action, optimal dosing and delivery strategies, as well as long-term safety and efficacy concerns in clinical settings.

#### 5.4.5 WJ-MSCs in treatment of diabetes mellitus

Diabetes mellitus is a group of metabolic diseases characterized by hyperglycemia due to deficiency in insulin secretion, insulin action, or both (Udler et al., 2017). The current cell therapy approach, i.e. islet transplantation, is challenging due to the limited donor availability, immune rejections and adverse effects of immunosuppressants (Bhonde et al., 2014). Therefore, utilizing MSCs’ secretome could be an effective intervention. In diabetes treatments, the mechanism of action of MSCs could be related to their ability to reside in pancreas and/or promoting repair by producing trophic factors including the growth factors, anti-inflammatory cytokines, and anti-oxidants (Chen et al., 2008; Karp and Leng Teo, 2009), all of which may exert anti-diabetic effects by modulating the immune system and by enhancing insulin sensitivity (English et al., 2010; Xie et al., 2016; Yin et al., 2018).

In type 2 diabetes (T2D) rodent model, WJ-MSCs injected intravenously through the tail vein were detected in several tissues including the lung, liver, spleen and pancreas, implying that the homing of WJ-MSCs was associated with recruitment to sites of tissue damage (Yin et al., 2018). Relative to UCB-MSCs and BM-MSCs, WJ-MSCs demonstrated a superior potential to differentiate into glucose stimulated insulin secreting (GSIS) cells and for better hyperglycemia control in type 1 diabetes (T1D) animal models (Chao et al., 2008; Wu et al., 2009; Wang et al., 2014; El-Demerdash et al., 2015). Interestingly, pancreatic islets co-cultured with umbilical cord blood (UCB)-MSCs induced a notable improvement in GSIS index and provided glycemic control post-transplantation in T1D mice model, supporting the notation that MSCs’ secretome enhanced the islet survival and function (Park et al., 2010; Keshtkar et al., 2020). In humans, a recent meta-analysis study assessing the therapeutic efficacy of WJ-MSCs and UCB-MSCs revealed a superior efficiency of the former cells for treating both types of diabetes mellitus (Kassem and Kamal, 2020). WJ-MSCs improved the glycemic control, β-cell function, decreased incidence of diabetic complications, and ameliorated the need for insulin injection in some of the patients (Kassem and Kamal, 2020). Furthermore, T1D patients treated with undifferentiated WJ-MSCs experienced a controlled postprandial plasma glucose levels and significant improvements in C-peptide and HbA1c levels during a 21-month follow-up period (Hu J. et al., 2013). In T2D patients, WJ-MSCs transplantation via intravenous and intrapancreatic endovascular injections retuned glycemic control and improved beta cell function by mechanisms that inhibited systemic inflammation and/or improved immunological regulation (Liu et al., 2014). Although, WJ-MSCs were used in these human clinical trials, yet most of the observed phenotypes were mainly due to the effects of their secretome (Fong et al., 2014).

## 6 WJ-MSCs and their microRNAs cargo

The human genome contains 1% microRNA (miRNA) coding genes, and around 30% of the protein coding genes are regulated by miRNAs (Gao et al., 2011). miRNAs are single-stranded, short (21–25 nucleotides), non-protein-coding RNAs that inhibit gene expression at post-transcriptional level by binding at the 3′ untranslated region of the target messenger RNA (mRNA) (Greco and Rameshwar, 2007; Lakshmipathy and Hart, 2008; Wagner et al., 2008; Aranda et al., 2009; Chen et al., 2010; Chen and Chen, 2011; Raut and Khanna, 2016). In most (80%) cases, this leads to the degradation of the mRNA or inhibition of protein translation (Sato et al., 2011; Dong et al., 2013; Raut and Khanna, 2016). In addition to containing proteins, WJ-MSCs’ exosomes harbor the coding (mRNAs) and non-coding (miRNAs) RNAs. In general, miRNA content of exosomes play a vital role in the biological function of exosomes and the source cells (Zhang et al., 2006; Gartel and Kandel, 2008; Gangaraju and Lin, 2009; Singh et al., 2013), by acting as signalosomes that can reprogram the cellular functions (Thomi et al., 2019a).

In general, miRNAs in stem cells have different-functions and play a significant role in determining fate of the cell. Stem cells exhibit the expression of specific miRNAs that are particularly associated with their distinct stages of differentiation (Greco and Rameshwar, 2007; Lakshmipathy et al., 2007; Bork et al., 2011; Tomé et al., 2011; Raut and Khanna, 2016). These characteristic expression signatures regulate the pluripotency and differentiation factors and can be used to characterize and monitor cell populations (Raut and Khanna, 2016). For example, an integrated analysis of miRNA and mRNA expression profiles of WJ-MSCs revealed 41 upregulated genes that represented the functions of WJ-MSCs (Chen et al., 2012). The key genes identified were KAL1 and PAPPA which are involved in maintaining the stemness of these cells, and regulate tissue development, cellular differentiation, and osteogenic protein signaling pathways (Bribián et al., 2006; Chapman et al., 2008). Moreover, the role of miRNA in determining a cell’s fate was confirmed by studying miRNA expression patterns during trans-differentiation of WJ-MSCs to hepatocyte-like cells (Raut and Khanna, 2016). The trans-differentiation of WJ-MSCs was initiated by treatment with histone deacetylase inhibitor and valproic acid and miRNA analysis revealed a significant upregulation of miRNAs involved in hepatic differentiation, including miR-23b cluster, miR-30a-5p, miR-26a-5p, miR-148a-3p, miR-192-5p, and miR-122-5p (Raut and Khanna, 2016). The targets of the upregulated miRNAs included pathways that block hepatic differentiation including transforming growth factor beta (TGFβ) and notch signaling pathways and those that inhibit the expression of transcription factors required to maintain the mesenchymal status (Li et al., 2011). Therefore, inhibition of these targets promoted the hepatic differentiation.

The miRNA expression patterns of WJ-MSCs EVs revealed miRNAs that were specific to WJ-MSCs EVs, along with those found in other types of stem cells (Zhou et al., 2018). They identified eight miRNAs that are common to WJ- and other MSCs-derived EVs, including miR-199a-3p, miR-24-3p, miR-29a-3p, miR-23a-3p, miR-638, miR-125b-5p, miR-630, and miR-21-5p (Zhou et al., 2018). The identified WJ-MSC-EV-specific miRNAs comprised of 25 miRNAs, including miR-144-3p and miR-142-3p for which biological activities have been documented (Zhou et al., 2018). miR-144-3p targets SMAD4, leading to negative regulation of osteogenic differentiation and proliferation of murine stem cells (Huang et al., 2016), while miR-142-3p promotes myeloid differentiation in hematopoietic stem cells, osteoblast differentiation in human fetal mesenchymal precursor cells, and erythroid differentiation in human embryonic stem cells (Wang XS. et al., 2012; Hu W. et al., 2013).

The therapeutic landscape of WJ-MSCs EVs, mediated by their cargo miRNAs, has shown neuroprotective and neuro-regenerative potential during hypoxic-ischemic injury (Joerger-Messerli et al., 2018). This protective and regenerative potential has been shown to be mediated by the let-7-5p miRNA family (let-7a and let-7e) that regulates caspase 3 activity (Joerger-Messerli et al., 2018). The potential of WJ-MSCs EVs to facilitate tissue repair was demonstrated by their ability to promote angiogenesis via the activation of the endogenous vascular endothelial growth factors (VEGF)-A expression (Chinnici et al., 2021). The EVs contained five miRNAs: miR-146a-5p, miR-27b-3p, miR-137, miR-125a-5p and miR-126-3p, which were upregulated and targeted the VEGF-A gene that is associated with angiogenesis (Chinnici et al., 2021). They also contained 15 miRNAs, including let-7b-5p, let-7e-5p, 21-5p, 99a-5p, 100-5p, 125b-5p, 127-3p, 145-5p, 193b-3p, 199a-3p, 214-3p, 221-3p, 222-3p, 320a, and 484, that were highly expressed in these EVs and exclusively targeted the thrombospondin 1 (THBS1) gene which is associated with the regulation of tissue repair (Chinnici et al., 2021). Moreover, WJ-MSCs-derived EVs were also found to promote the migration and proliferation of bone marrow-derived MSCs, chondrocytes, and M2 polarization of macrophages, eventually leading to osteochondral regeneration (Jiang et al., 2021). This effect was found to be promoted by 5 miRNAs (miR-92b, miR-29b, miR-374a, miR148a, and miR23a) in the EVs (Jiang et al., 2021).

In triple negative breast cancer (TNBC), WJ-MSCs-derived EVs were used to modify the cellular behavior and communication of TNBC cells and the non-cancer cells involved in tumorigenesis and metastasis (Chang et al., 2022). This effect was mediated by the internalization of WJ-MSCs EVs by the cells which resulted in the inhibition of tumor progression and metastasis (Chang et al., 2022). The transformation of the phenotypic characteristics is suggested to be mediated by the transfer of miRNA-125b from the WJ-MSCs EVs, which targets hypoxia-inducible factor 1-alpha (HIF1-α) and other genes related to proliferation, epithelial-mesenchymal transition, and angiogenesis (Chang et al., 2022). Therefore, analyzing the patterns of miRNA expression in WJ-MSCs EVs and characterizing their target genes and pathways can provide an insight into the therapeutic potential of WJ-MSCs. Similarly, as a novel method for the treatment of glioblastoma multiforme, WJ-MSCs’ exosomes were used to deliver miR-124, which reduced the expression of CDK6 and enhanced chemosensitivity to temozolomide, along with decreasing the migration of glioblastoma multiforme cells (Sharif et al., 2018).

Recent studies identified that a large amount of endogenous non-coding RNAs (ncRNAs) exist in MSCs which have critical regulatory effects on cell homeostasis and interaction with microenvironment, best reviewed in (Pant et al., 2021). Using an elegant transwell co-culture system, Sun et al., (2018) showed that circular RNA molecules were upregulated and secreted by WJ-MSCs in response to damaged endometrial stromal cells which improved the survival and repair of damaged endometrial cells. The authors reported a significant elevation in the expression levels of circRNA-8881-21, circRNA-0020492, circRNA-0026141, circRNA-4049-38, circRNA-0015825, circRNA-5028-15, and circRNA-0111659; as well as their host genes ASPM, MKI67, TROAP, WDR62, KIF14, and MYBL2, which were closely related to cellular proliferation, differentiation, and survival (Sun et al., 2018). Later, using the same experimental approach, the same research group reported the mechanistic role of circ6401-RNA, derived from WJ-MSCs secretome, in repairing the damaged endometrium by targeting miR26-b-1-5p, and hence upregulating the level of RAP1B which is a crucial angiogenic protein involved in the VEGF signaling pathway (Shi et al., 2020). Exosomes derived from WJ-MSCs, on the other hand, promoted the repair of myocardial infarction in rodent models and prevented ischemic cardiomyocytes apoptosis via the action of circ-0001273 RNA (Li et al., 2020).

## 7 Conclusion and future perspectives

Wharton’s jelly tissue in humans is an attractive source for MSCs. The isolated WJ-MSCs are of a naïve embryonic cell origin with a robust proliferation rate, reputable self-renewal rate, and multi-lineage differentiation potentials. In comparison with adult MSCs, WJ-MSCs are superior in respect of their minimal exposure to the environmental factors and genetic alteration, and by exhibiting better stemness characteristics. Therefore, the use of WJ-MSCs for clinical applications is ethically acceptable with minimal risk associated with the formation of teratoma (Zeddou et al., 2010; Al Madhoun et al., 2020).

WJ-MSCs’ secretome is enriched in bioactive molecules with the capacity to sustain cellular and tissue homeostasis. Owing to these dynamic characteristics, interventions using the WJ-MSCs’ secretome have been successful in treating inflammation, skin wounds, tumors, neurodegenerative disorders, tissue fibrosis, and diabetes. Therefore, it is noteworthy that WJ-MSCs’ secretome has tremendous potential, allowing for its allogeneic therapeutic applications. However, more detailed secretome profiling studies, especially those including proteomics and metabolomics, are required to gain a more in-depth understanding of its components and the underlying molecular mechanisms regulating their expression and secretion.

Noteworthy to mention that MSCs and WJ-MSCs cell-free therapeutic approach is a booming research field with tremendous potentials for novel clinical applications. There are several research publications and this review article may have covered only a limited number of the published research, yet we have overall reviewed, discussed and updated emerging research in the field to highlight importance and point to future directions.

Human WJ-MSCs and their secretome are a promising therapeutic modality for different diseases. Currently, *in vitro* and *in vivo* investigations have demonstrated the potential clinical benefits of these cells, in particular using the cell-free secretome for various clinical applications and avoiding the ethical concerns associated with cell transplantation. Although human clinical trials at phase I/II, for some diseases, are in progress, there remains a growing need for the longitudinal studies addressing the long-term efficacy of secretome-based cell-free therapy.

Indeed, WJ-MSCs and their secretome applications should follow the good manufacturing practice (GMP) guidelines for isolation, storage, quality assurance, and administration, all the while ensuring the safety and efficacy of its clinical applications. Future studies should focus on the cellular mechanisms and signaling pathways that could be exploited to enhance quality and benefits of secretome for a wide variety of biomedical applications. Importantly, an international society or organization should be on board to implement the safe practice of cell-free therapy.
